# Weiterbildung und Geringqualifizierung in der Digitalisierung – Ein Review zu Kontextfaktoren der Weiterbildungsbeteiligung Geringqualifizierter

**DOI:** 10.1007/s40955-022-00228-4

**Published:** 2022-11-17

**Authors:** Hadjar Mohajerzad, Luca Fliegener, Sophie Lacher

**Affiliations:** 1 Deutsches Institut für Erwachsenenbildung – Leibniz-Zentrum für Lebenslanges Lernen, Bonn, Deutschland; 2 Technische Universität Kaiserslautern, Kaiserslautern, Deutschland

**Keywords:** Geringqualifiziert, Weiterbildung, Weiterbildungsbeteiligung, Weiterbildungshemmnisse, Digitalisierung, Digitales Lernen, Covid-19, Scoping Review, Low-skilled, Continuing education, Participation in continuing education, Barriers to continuing education, Digitization, Digital learning, Covid-19, Scoping Review

## Abstract

Geringqualifizierte weisen im Vergleich zu anderen Qualifikationsgruppen die niedrigste Weiterbildungsbeteiligung auf. Vor dem Hintergrund der Digitalisierung und COVID-19-Pandemie wird Weiterbildung jedoch immer wichtiger für diese Zielgruppe. Mit Blick auf die betriebliche Weiterbildung wird eine soziale Selektivität von Geringqualifizierten deutlich. Mithilfe einer systematischen Literaturrecherche wurden Einflussfaktoren auf den Ebenen des Individuums, der Organisation, der Gesellschaft sowie der Politik auf die Weiterbildungsbeteiligung der Zielgruppe gesichtet. Insbesondere zeigt der Forschungsüberblick, dass häufiger quantitative Studien durchgeführt werden. Zugleich fehlt es noch an Studien, die institutionelle, organisationale und programmplanende Bedingungen untersuchen. Außerdem wurden während der COVID-19-Pandemie qualifikationsspezifische Unterschiede der Weiterbildungsbeteiligung deutlicher, woraus sich erste Indizien zur Weiterbildungsbeteiligung Geringqualifizierter unter Digitalisierungsbedingungen ableiten lassen.

## Abstract (en)

Low-skilled workers have the lowest participation in continuing education compared to other qualification groups. Against the backdrop of digitization, however, continuing education is becoming increasingly important for this target group. With a view to in-company continuing education, a social selectivity of the low-skilled becomes clear. In addition, factors at the individual, organizational, social and political levels have an impact on participation in continuing education. During the Covid 19 pandemic, qualification-specific differences in participation in continuing education became apparent, from which initial indications of the participation of low-skilled workers in continuing education under digitization conditions can be derived.

## Einleitung

Die Digitalisierung hält Einzug in die verschiedensten Lebensbereiche und beeinflusst diese nachhaltig, so auch die Arbeitswelt und die (Weiter‑)Bildung (Autorengruppe Bildungsberichterstattung 2020). Die digitale Transformation der Arbeitswelt schafft dabei neue Berufs- und Tätigkeitsfelder und nimmt Einfluss auf Kompetenzanforderungen von Arbeitnehmern und Arbeitnehmerinnen (ebd.). Insbesondere weisen Hilfs- und Fachkräftetätigkeiten ein höheres Substituierbarkeitspotenzial auf als Tätigkeiten, die eine höhere Qualifikation erfordern (Dengler und Matthes 2017). Dementsprechend wichtig werden Weiterbildungen, um Menschen auf den Wandel des Arbeitsmarktes vorzubereiten bzw. für veränderte Kompetenzbedarfe sowie -anforderungen zu befähigen und damit vor drohender Arbeitslosigkeit zu bewahren. Hierbei bildet das formale Bildungsniveau eine wichtige Voraussetzung für die individuellen Beschäftigungschancen (Hillmert 2017). Allerdings existiert weiterhin eine gesellschaftliche Gruppe von ca. 5 Mio. Menschen in Deutschland, die nur über eine geringe Qualifikation verfügt (Bertelsmann Stiftung 2018). Geringqualifizierte sind im Vergleich zu anderen Qualifikationsgruppen am stärksten von Arbeitslosigkeit sowie Armut bedroht (Reutter 2017; Bilger et al. 2017). Zudem lässt sich mit Blick auf die generelle Weiterbildungsbeteiligung feststellen, dass der Zugang zu Weiterbildung mit dem Qualifikationsniveau korreliert (Wotschack und Solga 2014). Dieses Phänomen wird als doppelte Selektivität (Faulstich 1981) oder als Weiterbildungsschere beschrieben (Schulenberg et al. 1978; Barz und Tippelt 2003), d. h. die Bildungsungleichheit wird durch Weiterbildung nicht ausgeglichen, sondern Weiterbildung hat einen verstärkenden Effekt, indem sie überwiegend von denjenigen in Anspruch genommen wird, die bereits über eine höhere formale Qualifikation verfügen („Matthäus-Effekt“). Dementsprechend kann Weiterbildung als sozial selektiv angesehen werden. Diese soziale Selektivität kann durch die Digitalisierung von Weiterbildung nochmals verstärkt werden, da hinsichtlich der Nutzung und des Zugangs zu digitalen Medien eine Ungleichheit in der Gesellschaft, entlang des Bildungsniveaus sowie des sozialen Status, zu erkennen ist (Autorengruppe Bildungsberichterstattung 2020). „Diese Disparitäten werden zu einer bildungspolitischen und gesellschaftlichen Herausforderung, wenn nicht sichergestellt wird, dass allen Adressatinnen und Adressaten von Bildungsangeboten gemäß ihrem individuellen Bedarf sowohl der Zugang zu digitalen Medien als auch der Aufbau entsprechender Kompetenzen ermöglicht wird“ (Autorengruppe Bildungsberichterstattung 2020, S. 297). Darüber hinaus liegt die Vermutung nahe, dass angesichts der in der Bevölkerung ungleich verteilten Nutzung der Zugänge zu digitalen Medien die Pandemie die Bildungschancen von marginalisierten Gruppen am härtesten trifft (z. B. Buddeberg und Stammer 2020; Gollob et al. 2021; Käpplinger und Lichte 2020). Personengruppen mit niedrigem Bildungsniveau, höherem Alter und geringerer Literalität profitieren kaum von digitalen Bildungsangeboten, da sie über weniger ausgeprägte Kompetenzen im Umgang mit digitalen Medien verfügen und digitale Bildungsangebote weniger nutzen, wie es in den Befunden zur digital divide oder digital inequality konstatiert wird (van Dijk 2012, 2020; Lutz 2019). Dabei sinkt das Risiko erwerbslos zu werden durch Aufstiegsfortbildungen, die durch die digitale Transformation der Arbeitswelt ebenfalls zunehmend an Bedeutung gewinnen (Bußmann und Seyda 2016).

Im Fokus des vorliegenden Beitrags stehen Geringqualifizierte aufgrund ihrer besonderen Bedrohung durch Arbeitslosigkeit und Ausschluss von gesellschaftlicher Teilhabe. Dabei wird ebenfalls berücksichtigt, welchen Einfluss die COVID-19-Pandemie auf die Weiterbildung der Zielgruppe nahm. Die COVID-19-Pandemie führte aufgrund der zeitweisen Einschränkung der Präsenzlehre zu einer schnelleren Digitalisierung des Bildungswesens und der Weiterbildung, wodurch Chancen und Risiken sowie Herausforderungen der Digitalisierung aufgezeigt wurden (Autorengruppe Bildungsberichterstattung 2020; Ehlert et al. 2021). So wurden im Jahr 2020 15 % der in Präsenz geplanten Weiterbildungsaktivtäten als Onlineformat durchgeführt, wohingegen fast die Hälfte (46 %) der online durchgeführten Weiterbildungsaktivitäten zuvor als Präsenzformat geplant waren und aufgrund der COVID-19-Pandemie umgestellt wurden (BMBF 2021). Ziel des Beitrags ist es, einen Überblick über den aktuellen Forschungsstand bezüglich der Weiterbildungsbeteiligung Geringqualifizierter vor dem Hintergrund der Digitalisierung zu geben. Dabei liegt ein Fokus auf dem Einfluss der COVID-19-Pandemie auf die Weiterbildungslandschaft Deutschlands sowie die Weiterbildungsbeteiligung Geringqualifizierter. Mittels einer systematischen Literaturrecherche erfasst die Studie systematisch die politischen, gesellschaftlichen, organisationalen sowie individuellen Einflussfaktoren der Weiterbildungsbeteiligung Geringqualifizierter vor dem Hintergrund der Digitalisierung und liefert damit eine Grundlage zur Analyse von Exklusionsmechanismen dieser Zielgruppe.

Die Argumentation folgt folgender Struktur: Zunächst wird der Begriff „Geringqualifiziert“ definiert. Daraufhin wird skizziert, welche Wirkung die Digitalisierung des Arbeitsmarktes auf die Gruppe der Geringqualifizierten hat und wie sich deren Weiterbildungsbeteiligung im Zuge der Digitalisierung entwickelt hat. Auf das methodische Vorgehen folgen die individuellen, organisationalen, politischen und gesellschaftlichen Einflussfaktoren, welche mittels des Mehrebenen-Angebots-Nachfragemodells (MAN-Modell) in Anlehnung an Boeren et al. (2010) dargestellt werden. Daraufhin wird der besondere Einfluss der COVID-19-Pandemie auf die Weiterbildungsbeteiligung Geringqualifizierter untersucht.

## Geringqualifizierte in Deutschland: Definition, Arbeitsmarkt und Weiterbildungsbeteiligung

### Definition Geringqualifiziert

Geringqualifiziert gilt als „Arbeitsmarktstatus“ (CEDEFOP 2018, S. 2), wird also immer in Bezug zu diesem definiert und ist somit eine relative Beschreibung. Zudem erfasst das Konzept Personen im erwerbsfähigen Alter, d. h. zwischen „15 bis 65 Jahre[n] (künftig bis 67 Jahre[n])“ (IAB-Forum – Institut für Arbeitsmarkt- und Berufsforschung der Bundesagentur für Arbeit 2017).

Der deutsche Weiterbildungsatlas definiert Geringqualifizierte anhand des formalen Bildungsniveaus: „Als geringqualifiziert gilt, wer über keinen berufsqualifizierenden Abschluss verfügt“ (Bertelsmann Stiftung 2018, S. 10). Auch in einer Studie des European Centre for the Development of Vocational Training (CEDEFOP) werden Geringqualifizierte ausschließlich aufgrund des Merkmals des Ausbildungsniveaus charakterisiert (CEDEFOP 2018, S. 2). Gleichzeitig üben die Autoren und Autorinnen Kritik an dieser Definition, die „das Konzept der Geringqualifizierung zu stark vereinfacht“ (ebd.) und fordern eine mehrdimensionale sowie dynamische Betrachtung der Kompetenzarten, Fähigkeiten und Faktoren, die ausschlaggebend für den Status ‚geringqualifiziert‘ am Arbeitsmarkt sind (ebd.). Die Spannweite und Komplexität des Konzepts der Geringqualifizierung wird bei der beispielhaften Betrachtung deutlich: So werden sowohl Menschen ohne Schulabschluss, Menschen ohne Hochschulabschluss, Menschen, deren (ausländische) Schul- und Berufsqualifikationen nicht anerkannt werden sowie Berufsentfremdete zu dieser Personengruppe gezählt (Bundesagentur für Arbeit 2021, S. 59).

Aufgrund der Heterogenität der Zielgruppe der Geringqualifizierten sowie der sich überschneidenden bzw. eine Beziehung aufweisenden Begriffe, wie ‚An- und Ungelernte‘, ‚Nicht formal Qualifizierte‘, ‚Funktionale Analphabet:innen‘, ‚Gering literalisierte Erwachsene‘ oder ‚Bildungsferne‘ wurde zunächst eine Annäherung an die heterogene Personengruppe der Geringqualifizierten angestrebt, indem bestehende Definitionen sowie verwandte Begriffe hinsichtlich der Geringqualifizierung untersucht wurden. Ziel war es, auf diesem Wege eine Basis für die systematische Literaturrecherche zu schaffen^1^. Als Ergebnis wurde folgende Definition abgeleitet, die das Konzept der Geringqualifizierung auf Basis der identifizierten Hauptaspekte bestehender Definitionen, den Erkenntnissen aus den Beziehungen zu bzw. Überschneidungen mit anderen Begriffen sowie aus einer relativen Perspektive heraus betrachtet:

Geringqualifizierte sind erwachsene Menschen, welche in Bezug auf konkrete berufliche Tätigkeitsbereiche zu einem bestimmten Zeitpunkt nicht oder nicht mehr über die als notwendig erachteten Kompetenzen verfügen. Die Bewertung als geringqualifiziert erfolgt dabei zum einen in Bezug auf das nationale durchschnittliche formale Bildungsniveau und zum anderen auf das Vorhandensein berufsqualifizierender Abschlüsse bzw. non-formal und informell erworbener Kompetenzen sowie deren zeitliche und inhaltliche Passung. Damit wird eine sehr heterogene Gruppe von Personen adressiert, welche:

a) über geringe mathematische und/oder schriftsprachliche und/oder „digitale“ Grundkompetenzen verfügen und/oder

b) über keinen formalen Berufsabschluss verfügen und/oder

c) über einen berufsqualifizierenden Abschluss verfügen, welcher am Arbeitsmarkt aktuell nicht nachgefragt wird und/oder

d) über keine oder unzureichende aktuell nachgefragten/geforderten beruflichen Kompetenzen verfügen (oder sich diese nicht kurzfristig aneignen können) und/oder

e) über berufliche Kompetenzen und/oder Qualifikationen verfügen, welche nicht formal anerkannt werden.

Geringqualifizierung ist damit eine anforderungsbezogene relative Beschreibung, welche zeitlich und in Bezug auf das berufliche Handlungsfeld und den Arbeitsmarkt differenziert und dynamisch bewertet werden muss.

Darüber hinaus zeigte sich in den Studien zur Weiterbildung, die diesem Forschungsbericht zugrunde liegen, dass in der Regel Personen zwischen 25 und 65 Jahren betrachtet wurden. Diese Altersspanne wird auch von der Arbeitsagentur genutzt und damit begründet, dass sich die Ausbildungsförderung an junge Erwachsene bis 25 Jahren richtet.^2^ Die Eingrenzung der Altersspanne sollte allerdings kritisch bei den vorliegenden Erkenntnissen reflektiert werden.

### Bedeutung der Veränderungen des Arbeitsmarktes für Geringqualifizierte

Die fortschreitende Digitalisierung nimmt Einfluss auf den Arbeitsmarkt sowie auf die arbeitsbezogenen Kompetenzanforderungen und verändert diese nachhaltig (Matthes und Severing 2017). Zudem betreffen die Digitalisierungs- und Automatisierungstendenzen der digitalen Transformation des Arbeitsmarktes verstärkt Tätigkeitsfelder mit vermehrt geringqualifizierten Beschäftigten, woraus sich ein hohes Substitutionspotential dieser Berufe ergibt (Dengler und Matthes 2017; Van Nieuwenhove und De Wever 2021). So weisen Helferberufe ein höheres Substitutionspotenzial (45 %) sowie einen stärkeren Anstieg dessen auf als Meister‑, Spezialisten- und Expertenberufe (Dengler und Matthes 2017). Nur Berufe des mittleren Qualifikationsrahmens verzeichnen in bestimmten Branchen ähnliche Substitutionspotentiale und könnten in Zukunft ebenfalls an Bedeutung verlieren (ebd.). Da Substitutionspotentiale in einem engen Zusammenhang mit dem Bildungsabschluss stehen, bilden Geringqualifizierte jedoch noch immer die am stärksten gefährdete Gruppe (ebd.). Demnach gehen als Folge der Veränderungen des Arbeitsmarktes auch Arbeitsplätze von Geringqualifizierten verloren (Funken und Schulz-Schaeffer 2008; Hirsch-Kreinsen und Ittermann 2017). Dies ist zum Teil darauf zurückzuführen, dass einfache repetitive Tätigkeiten entfallen und komplexere koordinative Tätigkeiten an Bedeutung gewinnen (Hirsch-Kreinsen und Ittermann 2017; Matthes und Weber 2019). Trotzdem können auch neue Arbeitsplätze entstehen, da die Besetzung von Tätigkeiten mit niedrigen Qualifikationsanforderungen kostengünstiger als die Automatisierung dieser sein könnte (Hirsch-Kreinsen und Ittermann 2017; Matthes und Weber 2019). Hieraus könnte ein neuer Weiterbildungsbedarf entstehen, um Geringqualifizierte für neue Tätigkeiten zu qualifizieren. Folglich führen die Veränderungen des Arbeitsmarktes aufgrund der steigenden Arbeits- und Kompetenzanforderungen zu einem steigenden Weiterbildungsbedarf Geringqualifizierter (Reutter 2017). In diesem Kontext gewinnt Weiterbildung an Bedeutung, um Menschen auf die verändernden Anforderungen des Arbeitsmarktes vorzubereiten und vor drohender Arbeitslosigkeit zu schützen (ebd.).

Insgesamt kann festgehalten werden, dass das Anforderungsniveau der Tätigkeit, das Berufssegment sowie das Bildungsniveau entscheidende Faktoren bezüglich der Substituierbarkeit von Berufen sind (Dengler und Matthes 2017). Dementsprechend ist die Gruppe der Geringqualifizierten stärker als andere Qualifikationsgruppen von Substitutionen im Zuge der Digitalisierung und Automatisierung betroffen, am stärksten von Arbeitslosigkeit bedroht und am häufigsten in atypischen Verhältnissen angestellt (Sperber und Walwei 2017). Die COVID-19-Pandemie verstärkte die herausfordernde Arbeitsmarktsituation Geringqualifizierter nochmals. So wiesen Beschäftigte auf Helferniveau zu Beginn der Pandemie neben Selbstständigen das größte Risiko eines Arbeitsplatzverlustes auf (Statistisches Bundesamt et al. 2021). Insgesamt waren Helfertätigkeiten auf doppelte Weise durch die Krise betroffen, indem der Pandemieausbruch die alljährliche Beschäftigungszunahme im Frühling einschränkte und Bereiche mit vielen Helfertätigkeiten, wie das Gastgewerbe, unter einem starken Beschäftigungseinbruch litten (Seibert et al. 2021).

### Die Weiterbildungsbeteiligung Geringqualifizierter

Auf Basis der Daten des Adult Education Survey (AES) des Bundesministeriums für Bildung und Forschung (BMBF) liegt die Weiterbildungsbeteiligung Geringqualifizierter hinter derer aller anderen Qualifikationsgruppen (Seyda 2019b). Trotzdem verzeichnete diese Zielgruppe im Zeitraum von 1979 bis 2018 einen stärkeren Anstieg der Weiterbildungsbeteiligung als die anderen Qualifikationsgruppen und konnte sich der Gruppe der Akademiker und Akademikerinnen annähern (ebd.). Dennoch nehmen Personen ohne abgeschlossene Berufsausbildung im Vergleich zu anderen Qualifikationsgruppen deutlich seltener an Weitbildungen teil (Martin und Rüber 2016; Ramos und Harris 2012). Ein Überblick über die Gründe für die geringe Teilnahme bzw. die Nicht-Teilnahme an Weiterbildung ist bereits in den 1980er-Jahren eruiert worden (Cross 1992), viele quantitative Studien schlossen sich hieran an. Die Gründe für die geringe Weiterbildungsteilnahme Geringqualifizierter sind vielzählig, so weisen geringqualifizierte Beschäftigungen seltener Weiterbildungsangebote auf und arbeiten häufig in kleinen sowie mittleren Unternehmen, die weniger Weiterbildungen anbieten als größere Unternehmen (Ziegler und Akbar 2021). Zudem sehen Geringqualifizierte mehr Hinderungsgründe bei Weiterbildungen, aus denen eine Nicht-Teilnahme resultiert, als andere Qualifikationsgruppen (Osiander 2019). Auch bei den Gründen der Nicht-Teilnahme werden qualifikationsspezifische Unterschiede deutlich, so herrscht bei Geringqualifizierten eine größere Unsicherheit, ob sich Weiterbildungen auszahlen, sie sehen sich als ausreichend qualifiziert und eher als lernentwöhnt an (ebd.). Hinzu kommt, dass Geringqualifizierte im Vergleich zu Nichtgeringqualifizierten eine höhere Abbruchquote bei Maßnahmen zur Förderung beruflicher Weiterbildung aufweisen (ebd.).

Ein Blick auf das betrieblich organisierte Weiterbildungsangebot – als größtes Weiterbildungssegment Deutschlands (Autorengruppe Bildungsberichterstattung 2020) – zeigt, dass Geringqualifizierte weniger Weiterbildungsangebote sowie eine niedrigere Weiterbildungsbeteiligung aufweisen als andere Qualifikationsgruppen (Matthes und Weber 2019; Pfeiffer et al. 2019) und dass Betriebe sozial selektiv weiterbilden (Eichhorst und Schroeder 2019). Es werden hauptsächlich die Stammbelegschaft, hochqualifizierte Beschäftigte sowie Führungskräfte weitergebildet (ebd.). Trotzdem zeigt sich, dass zielgruppenorientierte Weiterbildungsangebote für Geringqualifizierte zugenommen haben (Schöpper-Grabe und Vahlhaus 2019). Da sich überproportional viele Geringqualifizierte im Rechtskreis des Sozialgesetzbuches II (SGB II) befinden und diese aufgrund ihrer Erwerbslosigkeit von betrieblichen Weiterbildungsangeboten ausgeschlossen werden, findet allerdings auch hier eine Selektion statt, die sich negativ auf die Weiterbildungsbeteiligung der Zielgruppe auswirkt (Seyda 2019b).

## Methodisches Vorgehen

Im Oktober 2021 wurde ein erster Durchgang der systematischen Literaturrecherche durchgeführt, auf die Mitte Dezember 2021 eine weitere Recherche zur Identifizierung von Kontextfaktoren folgte. Der Sichtung und Analyse der Publikationen lag die leitende Forschungsfrage zugrunde: *Welche individuellen, organisationalen, (förder-)politischen und gesellschaftlichen Faktoren bedingen, dass die Weiterbildungsbeteiligung Geringqualifizierter (nicht) gelingt?* Im Rahmen dessen wurde auch der besondere Einfluss der COVID-19-Pandemie auf die Weiterbildungsanbieter sowie auf die Weiterbildungsbeteiligung der Zielgruppe dargestellt. Hierfür wurde der Zeitraum 2016 bis 2021 gewählt, da hier Kontextfaktoren sowohl vor als auch während der Pandemie eruiert werden konnten. Um die Charakteristika der Weiterbildungsbeteiligung Geringqualifizierter festzustellen, wurde die Literaturrecherche nach dem Vorgehen eines Scoping Reviews durchgeführt. Scoping Reviews werden insbesondere dann verwendet, wenn das Ziel des Reviews die Ermittlung von Wissenslücken und die Erklärung von Kontextfaktoren ist (Munn et al. 2018). Außerdem dienen Scoping Reviews der Orientierung über den Stand der (Forschungs‑)Literatur sowie über Art und Umfang der Forschungsergebnisse (Grant und Booth 2009). Tab. 1 enthält eine Zusammenfassung der Recherchestrategie. Die Suchbegriffe wurden auf Basis einer vorangegangenen Literaturrecherche entwickelt. Da der Fokus auf Geringqualifizierte in Deutschland liegt, erfolgte die Recherche in deutschsprachigen Datenbanken (Fachinformationssystem Bildung und Pollux).

**Table 1:** 

Datenbank	Kombination von Suchbegriffen pro Suchdurchlauf				
	Erste Suchbegriffe		Zweite Suchbegriffe		Dritte Suchbegriffe
Fachinformationssystem Bildung (FIS)^a^ Pollux	Geringqualifiziert* Ungelernter Arbeitnehmer Niedrig Qualifizierter niedrigqualifiziert	UND	Programm Weiterbildung Qualifizierungsmaßnahme Corona Weiterbildungsangebot Digitalisierung Kompetenz Teilnahme Digitalisierung	–	–
	Weiterbildung	UND			
	Weiterbildungsangebot	UND	Corona		
	Beteiligung	UND	Weiterbildung		
Fachinformationssystem Bildung (FIS)^b^	Digital* Medien* Internet* E‑Learning Blended Learning MOOC Online* Technologie Computer* Corona Covid Pandemie	UND	Organisation* Einrichtung Institution	UND	Geringqualifiziert* gering Qualifizierte geringe Qualifikation Angelernt* Ungelernt* Niedrigqualifiziert* Niedrige Qualifikation gering literalisiert* geringe Literalität Bildungsferne Funktionale Analphabeten funktionaler Analphabetismus

^a^FIS-Bildung ermöglicht eine Stichwortsuche über verschiedene Suchfelder. So wurden im ersten Durchgang jeweils alle dargestellten Begriffe innerhalb einer Suche im Suchfeld „Freitext“ und im Zeitraum von 2016 bis 2021 recherchiert. Den gewählten Schlagwörtern liegt die Systematik von FIS Bildung zugrunde. Die politikwissenschaftliche Datenbank Pollux bietet in ihrer erweiterten Suchfunktion ebenfalls die Möglichkeit, die dargestellten Begriffe mit AND zu verbinden sowie den Suchzeitraum von 2016 bis 2021 zu definieren. Im Rahmen der Recherche wurden erneut alle dargestellten Begriffe innerhalb einer Suche miteinander kombiniert

^b^Im zweiten Durchgang wurde der Kontext Organisation zusätzlich mitberücksichtigt. Dabei wurden die ersten, zweiten und dritten Suchbegriffe im Suchfeld „Schlagwort“, dann im Suchfeld „Schlagwort“ sowie im Suchfeld „Titel“ recherchiert. Außerdem wurden die zweiten und dritten Suchbegriffe ohne den ersten Suchbegriff im Suchfeld „Schlagwort“ und im Suchfeld „Titel“ gesichtet

**Figure 1:**
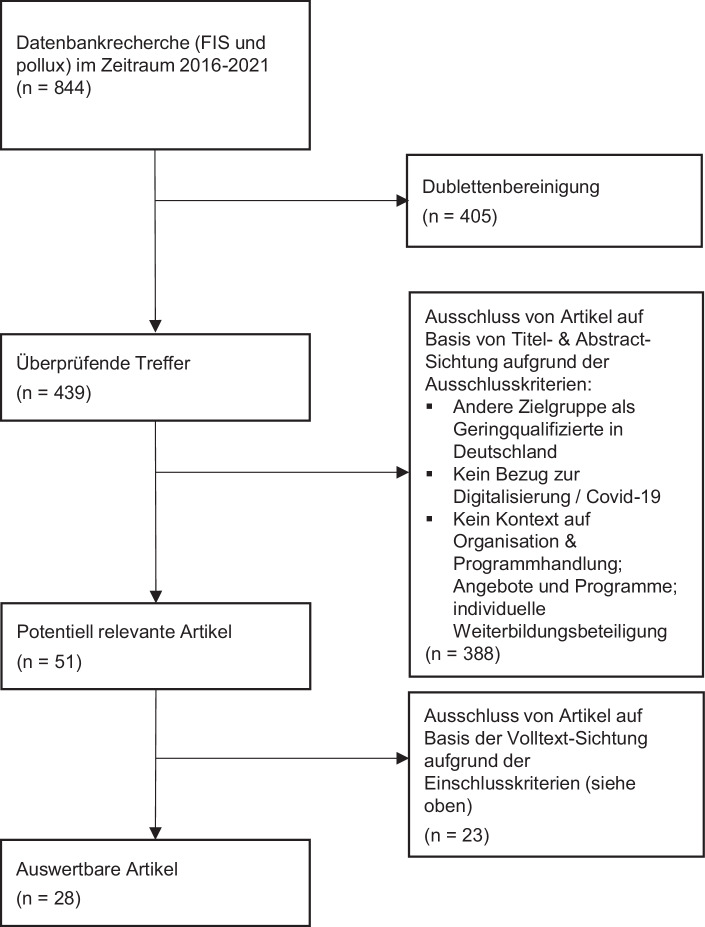

Im Zeitraum von 2016 bis 2021 wurden insgesamt 844 Publikationen ermittelt (s. Abb. 1). Nach der Reduktion um Dubletten blieben 439 Publikationen übrig. In einem nächsten Schritt wurden graue Literatur (Konferenzberichte, (unveröffentlichte) Dissertationen, Praxishandbücher) und Veröffentlichungen, deren Abstracts keine Relevanz für die Fragestellung, z. B. kein Fokus auf Digitalisierung, Geringqualifizierte und/oder Weiterbildung aufwiesen, ausgeschlossen. Schließlich lagen nach der Volltextsichtung 51 Publikationen vor. Weitere 23 Publikationen wurden schlussendlich ausgeschlossen (s. Abb. 1), da deren Handlungskontexte sich entweder auf Geringqualifizierte oder Weiterbildung, nicht aber auf Weiterbildungsbeteiligung und -anforderung Geringqualifizierter bezogen.

## Ergebnisse

### Beschreibung des Corpus

Von den 28 identifizierten Veröffentlichungen sind 13 Artikel keine empirischen Studien (Autorengruppe Bildungsberichterstattung 2020; BMAS – Bundesministerium für Arbeit und Soziales und BMBF – Bundesministerium für Bildung und Forschung 2019; Bundesinstitut für Berufsbildung 2020; Bundesregierung 2021; Eichhorst und Schroeder 2019; Hofmann et al. 2018; Klaus et al. 2020; Klös 2021; Osiander 2019; Schmidt-Hertha 2021; Seyda 2019a, b; Umsetzungsbericht der NWS in: BMAS – Bundesministerium für Arbeit und Soziales und BMBF – Bundesministerium für Bildung und Forschung 2021). Von diesen wurden in vier Artikeln auf deskriptive Auswertungen aus den Statistiken der Bundesagentur für Arbeit (BA) (Bundesregierung 2021; Hofmann et al. 2018; Klaus et al. 2020; Osiander 2019) Bezug genommen und in einem Artikel deskriptive Befunde zur Bestandaufnahme in Deutschland Autorengruppe Bildungsberichterstattung (2020) erstellt. Bei den empirischen Studien handelt es sich in 11 Artikeln um Daten aus Sekundäranalysen des Nationalen Bildungspanels – NEPS (Ehlert et al. 2021; Kleinert et al. 2020, 2021; Rüber und Widany 2021), des Sozio-oekonomischen Panels – SOEP (Rüber und Widany 2021), des Adult Education Surveys – AES (Rüber und Widany 2021), des European Company Surveys – ECS (Wiß 2017), der Volkshochschulstatistik (Rüber und Widany 2021), des IAB-Betriebspanels (Seyda et al. 2018; Wotschack 2017, 2020), des wb-monitors (Christ et al. 2021), des Instituts der deutschen Wirtschaft Personalpanels – IW (Schöpper-Grabe und Vahlhaus 2019) sowie des Programme for the International Assessment of Adult Competencies – PIAAC (Hornberg et al. 2021). In diesen Artikeln werden in allen 11 empirischen Studien statistische Auswertungen berichtet. Überwiegend werden deskriptive Statistiken und einfache Korrelationen berichtet, in sechs Artikeln werden multivariate bzw. komplexere Inferenzstatistiken (Ehlert et al. 2021; Kleinert et al. 2020, 2021; Wiß 2017; Wotschack 2017, 2020) durchgeführt. In vier Artikeln werden qualitative (inhaltsanalytische) Auswertungen realisiert (Grebe et al. 2017; Hermeling 2017; Pfeiffer et al. 2019; Wolf et al. 2018). Von diesen wurden in zwei Artikeln auch standardisierte Befragungen innerhalb der Untersuchung miteinbezogen (Grebe et al. 2017; Hermeling 2017). Die hier untersuchten Artikel weisen damit tendenziell Beschreibungswissen zum Ist-Zustand auf und bieten eher wenig Erklärungs- oder Verbesserungswissen.

### Einflussfaktoren auf die Weiterbildungsbeteiligung Geringqualifizierter

**Figure 2:**
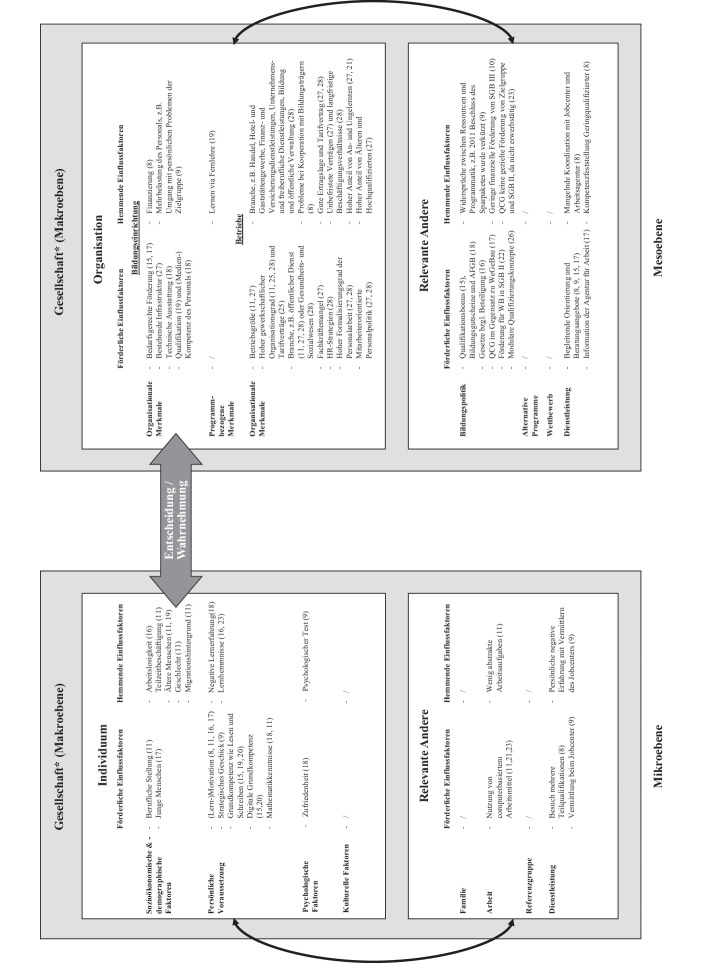

Zur Verortung der förderlichen und hemmenden Einflussfaktoren wurde die Grundheuristik des MAN-Modells herangezogen. Boeren et al. (2010) konzeptualisieren mit diesem Modell potenzielle Einflussfaktoren unterschiedlicher analytischer Ebenen (Mikro-, Meso- und Makroebene). Insgesamt wurden 81 Einflussfaktoren in den 28 Artikeln identifiziert, die, inhaltlich nach dem Nachfrage-Angebotsmodell, in 63 verschiedene positive und negative Einflussfaktoren gebündelt wurden (Abb. 2). In der Abb. 2 fallen Lücken bei den Feldern zum Individuum („kulturelle Faktoren“, „Familie“ und „Referenzgruppe“) sowie der Mesoebene („programmbezogene Merkmale“, „alternative Programme“ und „Wettbewerb“) auf. Insbesondere im Feld der Mesoebene wird eine geringe Empirie zur Untersuchung von Einflussfaktoren zu den programmbezogenen Merkmalen deutlich. Im Folgenden werden die Einflussfaktoren vorgestellt:

Auf der **Ebene des Individuums** wurden sowohl förderliche als auch hemmende Einflussfaktoren bezüglich der Weiterbildungsteilnahme von Geringqualifizierten gesichtet (Abb. 2). Durch die Digitalisierung steigen Arbeits- und Kompetenzanforderungen gegenüber Geringqualifizierten (Eichhorst und Schroeder 2019; Seyda et al. 2018), somit wirken sich insbesondere persönliche Voraussetzungen und Kontextfaktoren der Arbeit förderlich auf deren Weiterbildungsbeteiligung aus (Hornberg et al. 2021; Seyda et al. 2018; Seyda 2019b). Des Weiteren nehmen Arbeitsbedingungen (Möglichkeit zu Homeoffice, Systemrelevanz und Arbeitszeitveränderungen) Einfluss auf die Weiterbildungsbeteiligung, dabei arbeiten Geringqualifizierte oftmals unter für die Weiterbildungsbeteiligung nicht förderlichen Arbeitsbedingungen (Ehlert et al. 2021; Kleinert et al. 2020). Grundbildung wird immer wichtiger für die Bewältigung des Arbeitsprozesses und für eine erfolgreiche Weiterbildung (Schöpper-Grabe und Vahlhaus 2019). Dementsprechend bildet eine mangelnde Grundbildung eine Hürde der Weiterbildungsbeteiligung. Betriebe bieten vermehrt Weiterbildungen zu arbeitsplatzbezogener Grundbildung an und weisen auf einen steigenden Bedarf hin (Klös 2021; Schmidt-Hertha 2021; Schöpper-Grabe und Vahlhaus 2019). Trotzdem sind diese meist tätigkeitsorientiert, werden erst durchgeführt, wenn Weiterbildungen für die Erledigung der Arbeit nötig werden und vermitteln keine allgemeine Grundbildung (Schöpper-Grabe und Vahlhaus 2019). Diese wäre jedoch wichtig, da eine mangelnde Grundbildung eine zusätzliche Hürde der Weiterbildungsbeteiligung Geringqualifizierter bildet und eine vorhandene Grundbildung die Beschäftigungssicherheit erhöht, zumal dies die Basis für das erfolgreiche Partizipieren an weiteren Weiterbildungen bildet (Schöpper-Grabe und Vahlhaus 2019). Der Erhalt der Weiterbildungsfähigkeit und die Partizipation an Weiterbildung, vor dem Hintergrund des Lebenslangen Lernens, werden aufgrund der sich ändernden Arbeitsanforderungen von immer größerer Bedeutung. Auf der Ebene des Individuums nehmen auch soziodemografische Faktoren Einfluss auf die Weiterbildungsbeteiligung von Geringqualifizierten, so bilden sich tendenziell eher Personen ohne Migrationshintergrund weiter, meist sind diese eher älter und weiblich (Hornberg et al. 2021).

Auf der **Ebene der Organisation** wird im MAN-Modell durch die Literaturrecherche eine zusätzliche Unterscheidung zwischen Bildungseinrichtungen und Betrieben vorgenommen. Insgesamt wurden acht Einflussfaktoren auf der Ebene der Bildungseinrichtungen genannt. Dabei werden drei Einflussfaktoren des Personals in den Artikeln berichtet. Insbesondere sind betriebliche Weiterbildungsangebote für Geringqualifizierte selten zugänglich (Wotschack 2020) und Betriebe bilden sozial selektiv weiter (hauptsächlich die Stammbelegschaft, höher Qualifizierte sowie Führungskräfte) (Eichhorst und Schroeder 2019). Somit werden Geringqualifizierte systematisch benachteiligt. Ist der Anteil von An- und Ungelernten, Älteren und Hochqualifizierten in den Betrieben hoch, so nehmen Geringqualifizierte seltener an Weiterbildungen teil (Wotschack 2017). Während in Branchen wie Handel, Hotel- und Gaststättengewerbe, Finanz- sowie Versicherungsdienstleistungen, Unternehmens- und freiberufliche Dienstleistungen, Bildung und öffentliche Verwaltung aus den Bereichen Bergbau, Energie, Wasser und Abfall, ein hemmender Einfluss auf die Weiterbildungsbeteiligung Geringqualifizierter berichtet wird (Wotschack 2020), werden in Branchen wie dem öffentlichen Dienst, Personen im öffentlichen Recht, und dem Gesundheits- sowie Sozialwesen die Weiterbildungsbeteiligung Geringqualifizierter eher gefördert (Hornberg et al. 2021; Wiß 2017; Wotschack 2020). Veränderungen im Arbeitsumfeld (Einführung neuer Computerprogramme, neue/veränderte Dienstleistungen sowie neue direkte Vorgesetzte) haben ebenfalls einen positiven Einfluss auf die Weiterbildungsbeteiligung Geringqualifizierter (Seyda et al. 2018). Demnach kann der Anstieg der Weiterbildungsbeteiligung Geringqualifizierter zum Teil durch die Einführung neuer Technologien im Arbeitsprozess erklärt werden (Seyda et al. 2018; Seyda 2019a). Trotzdem wird deutlich, dass Geringqualifizierte im Vergleich zu allen anderen Beschäftigten seltener durch digitale Medien und webbasierte Weiterbildungen geschult werden (Schöpper-Grabe und Vahlhaus 2019). Aus betrieblicher Sicht ist das selbstgesteuerte Lernen mittels digitaler Medien am wenigsten für Geringqualifizierte geeignet, daher wird mit Blick auf die Zielgruppe ein Blended-Learning-Ansatz mit Lernprozessbegleitern (Schöpper-Grabe und Vahlhaus 2019; Seyda 2019b) sowie die Schaffung weiterbildungsförderlicher Arbeitsplätze (Seyda 2019b) empfohlen. Zusammenfassend zeigt sich, dass die Weiterbildungsbeteiligung Geringqualifizierter – auch unter Berücksichtigung der Arbeitsanforderungen sowie des Arbeitsumfelds – noch hinter allen anderen Qualifikationsgruppen liegt. Der Unterschied zwischen den Qualifikationsgruppen ist dabei insbesondere auf individuelle und betriebliche Faktoren zurückzuführen.

**Bildungspolitische Programme und Gesetze**, wie der Qualifikationsbonus (Klös 2021), Bildungsgutscheine, das Aufstiegsfortbildungsförderungsgesetz (Rüber und Widany 2021), modulare Qualifizierungskonzepte (Wolf et al. 2018) und das Qualifikationschancengesetz (QCG) (Bundesregierung 2021; Pfeiffer et al. 2019) sind für die Weiterbildungsbeteiligung von Geringqualifizierten förderlich (Bundesregierung 2021; Osiander 2019). Zudem wird die Weiterbildung Geringqualifizierter in der Nationalen Weiterbildungsstrategie (NWS) gefördert (BMAS – Bundesministerium für Arbeit und Soziales und BMBF – Bundesministerium für Bildung und Forschung 2019; Umsetzungsbericht der NWS in: BMAS – Bundesministerium für Arbeit und Soziales und BMBF – Bundesministerium für Bildung und Forschung 2021). Zwar sind Bildungsgutscheine und das Aufstiegsfortbildungsförderungsgesetz förderlich für die Weiterbildungsbeteiligung Geringqualifizierter, aber sie fokussieren nur finanzielle Hürden – im Falle der Bildungsgutscheine – für Menschen mit niedrigem Einkommen und – im Falle des Aufstiegsfortbildungsförderungsgesetzes – für Menschen mit bereits vorhandenem Abschluss (Rüber und Widany 2021). Diese Fördermöglichkeiten zielen nicht auf die heterogene Gruppe der Geringqualifizierten, sondern nur auf Teile der Gruppe. Das QCG ist förderlicher für die Weiterbildungsbeteiligung Geringqualifizierter als das Programm Weiterbildung Geringqualifizierter und beschäftigter älterer Arbeitnehmer in Unternehmen (WeGebAU), da das QCG eine flächendeckende Förderung unabhängig von Qualifikationen vorsieht (Klaus et al. 2020; Pfeiffer et al. 2019). Trotzdem ist die Förderung des QCG im Vergleich zum WeGebAU-Programm hinsichtlich der Zielgruppe unspezifischer, wodurch das Ziel, hauptsächlich Personen ohne Berufsabschluss zu fördern, in Teilen verfehlt wird (Seyda 2019b). Jedoch kann die Möglichkeit der allgemeinen, nicht-zielgruppenbezogenen Weiterbildung zu Mitnahmeeffekten führen (Seyda 2019b). Die Wirksamkeit des Arbeit-von-morgen-Gesetzes kann noch nicht durch Statistiken bewertet werden, da es erst am 01.10.2020 in Kraft getreten ist. Die oben aufgeführten Gesetze und Programme richten sich nur an erwerbstätige Menschen, nicht an Arbeitslose (Eichhorst und Schroeder 2019). Da ein überproportionaler Teil an Arbeitslosen im SGB II geringqualifiziert ist, haben diese keinen Zugang zu den Förderprogrammen und -gesetzen, weshalb für diese Gruppe eine aktive Weiterbildungsförderung durch abschlussbezogene Weiterbildungen empfohlen wird (Hofmann et al. 2018; Klös 2021; Seyda 2019a). Modulare Nachqualifizierungen zeigen einige Vorteile für Geringqualifizierte, da individuelle motivationsbedingte Barrieren abgebaut, individuelle Vorerfahrungen berücksichtigt und Bildungsphasen an Lebensumstände angepasst werden. Dies könnte die Weiterbildungsbeteiligung lernentwöhnter Gruppen verbessern (Hofmann et al. 2018; Klös 2021; Wolf et al. 2018). Geringqualifizierte haben seit 2020 einen Rechtsanspruch auf die Förderung einer berufsabschlussbezogenen Weiterbildung (Wolf et al. 2018). Dies schafft zwar keine neuen Förderbestände, kann aber als eine Stärkung der individuellen Verhandlungsposition gegenüber der entscheidenden Stelle gesehen werden (Seyda 2019a).

Schließlich werden in den Artikeln **gesellschaftliche Einflussfaktoren** wie der digitale Strukturwandel und die technische Veränderung als förderlich für die Weiterbildungsbeteiligung Geringqualifizierter berichtet (Pfeiffer et al. 2019; Seyda 2019b), während die COVID-19-Pandemie (Ehlert et al. 2021; Kleinert et al. 2021) und die Heterogenität der Gesellschaft, sowie die Tatsache, dass Gesellschaften aus unterschiedlichen sozialen Gruppen bestehen, als hemmende Einflussfaktoren beschrieben werden (Rüber und Widany 2021). Besonders die COVID-19-Pandemie führte zu erheblichen Auswirkungen auf die Weiterbildungsbeteiligung (Ehlert et al. 2021; Kleinert et al. 2021) und die Weiterbildungsanbieter (Bundesinstitut für Berufsbildung 2020; Christ et al. 2021) sowie auf die Inanspruchnahme förderpolitischer Gesetze (Bundesregierung 2021).

### Auswirkungen der COVID-19-Pandemie

Die COVID-19-Pandemie bewirkte aufgrund der zeitweisen Aussetzung der Präsenzlehre, der vorher häufigsten Form von Lehrveranstaltungen, zu einer Beschleunigung der Digitalisierung des Weiterbildungssektors (Autorengruppe Bildungsberichterstattung 2020). Dies hatte einen Digitalisierungsschub in der Durchführung von Lehrveranstaltungen zur Folge, da Veranstaltungen, die nicht ausfielen, im digitalen Raum fortgesetzt werden mussten (Christ et al. 2021). Dadurch wird es ermöglicht, die Auswirkungen der Digitalisierung von Weiterbildung auf Weiterbildungsanbieter (Abschn. 4.3.1) sowie auf die Weiterbildungsbeteiligung Geringqualifizierter (Abschn. 4.3.2) zu betrachten. Damit können Rückschlüsse darauf gezogen werden, inwieweit eine Digitalisierung von Weiterbildung die Weiterbildungsteilnahme Geringqualifizierter beeinflusst hat.

#### Auswirkungen der COVID-19-Pandemie auf die Weiterbildungsanbieter

Im Jahr 2020 hat die COVID-19-Pandemie bei 44 % der Weiterbildungsaktivitäten für Veränderungen gesorgt, dabei wurden 24 % der Formate mit Hygienebestimmungen durchgeführt, 15 % als Onlineformat statt in Präsenz und 10 % unterbrochen oder verschoben (BMBF 2021). Der Wechsel von Präsenz- zu Onlineformaten während des ersten bundesweiten Lockdowns stellte Weiterbildungsinstitutionen vor große Herausforderungen (Christ et al. 2021). Dabei wurden Diskrepanzen zwischen verschiedenen Typen von Weiterbildungsanbietern hinsichtlich des Realisierungsgrades von Veranstaltungen sowie dem Anteil an digital durchgeführten Veranstaltungen deutlich, die nach Einschätzung der Anbieter auch nach dem Lockdown bestehen blieben (ebd.). Diese Unterschiede lassen sich mit dem Digitalisierungsgrad sowie der Angebotsstruktur der verschiedenen Institutionstypen erklären (ebd.). Weiterbildungsanbieter, die sich eher an Hochgebildete richteten, wiesen einen höheren Digitalisierungsgrad und auch eine höhere Rate an realisierten Weiterbildungen auf (ebd.). Dementsprechend kann davon ausgegangen werden, dass der Coronalockdown die Weiterbildungsangebote für Geringqualifizierte stärker eingeschränkt hat als für Hochqualifizierte, wobei die Unterschiede der Einschränkungen auch nach dem Ende des ersten Coronalockdowns zum Teil bestehen blieben.

#### Auswirkungen der COVID-19-Pandemie auf die Weiterbildungsbeteiligung Geringqualifizierter

Im ersten coronabedingten Lockdown 2020 wurden digitale Weiterbildungsangebote häufiger zu beruflichen Zwecken genutzt und weniger zu privaten (Ehlert et al. 2021; Kleinert et al. 2021). Dies könnte darauf zurückzuführen sein, dass die COVID-19-Pandemie zu einer verbreiterten Verwendung von Technologien geführt hat, für deren Nutzung neues Wissen angeeignet werden musste (Kleinert et al. 2021). Differenziert nach Qualifikationsgruppen nutzten Erwerbstätige ohne Schulabschluss mehr digitale berufliche Weiterbildungsangebote als im Jahr zuvor und verzeichneten im Frühjahr 2020 eine höhere Nutzungsrate als Erwerbstätige mit Berufsabschluss (Ehlert et al. 2021; Kleinert et al. 2021). Aufgrund der geringen Stichprobe und der großen Konfidenzintervalle ist dieses Ergebnis jedoch mit Vorsicht zu betrachten (Ehlert et al. 2021). Erwerbstätige mit Hochschulabschluss wiesen die höchste Nutzungsrate auf (ebd.). Dementsprechend blieben die Unterschiede in der Weiterbildungsbeteiligung zwischen Hoch- und Niedriggebildeten bestehen, unter Kontrolle soziodemographischer Faktoren wird eine größere Polarisierung deutlich (ebd.). Dies ist zum Teil auf Veränderungen von Arbeitsbedingungen (Homeoffice, Arbeitszeitveränderung, Systemrelevanz) im Zuge der Coronapandemie zurückzuführen (ebd.). Die Möglichkeit zur Arbeit im Homeoffice hatte auch schon vor der Coronapandemie einen positiven Effekt auf die Nutzungsrate digitaler Lernangebote, durch die Pandemie wurde mehr Beschäftigten die Möglichkeit dazu gegeben (Ehlert et al. 2021; Kleinert et al. 2021). Gleichzeitig lernten Beschäftigte ohne die Möglichkeit zur Arbeit im Homeoffice während der Pandemie seltener digital (ebd.). Somit waren Geringqualifizierte seltener als Hochqualifizierte veränderten Arbeitsbedingungen ausgesetzt, die sich förderlich auf die Weiterbildungsbeteiligung auswirkten (ebd.). Die systematischen Unterschiede zwischen diesen zwei Gruppen hinsichtlich der Arbeitsbedingungen haben sich in den ersten Monaten der Coronapandemie nochmals verstärkt (Kleinert et al. 2020). Dementsprechend hatte die Coronapandemie, durch die qualifikationsabhängige Veränderung von Arbeitsbedingungen, einen indirekten Einfluss auf die Weiterbildungsbeteiligung (Ehlert et al. 2021; Kleinert et al. 2021). Zudem liegt die Vermutung nahe, dass Unterschiede der Weiterbildungsbeteiligung zwischen Niedrig- und Hochgebildeten über die Pandemie hinweg bestehen blieben und sich gegebenenfalls aufgrund der im verschiedenen Maß veränderten Arbeitsbedingungen der Qualifizierungsgruppen verfestigt haben (ebd.). Dies würde gegen die Annahme sprechen, dass digitale Angebote aufgrund des vereinfachten Zugangs sowie der zeitlichen und örtlichen Flexibilität niedrigschwelliger sind und dadurch die Teilnahme an Weiterbildung vereinfacht wird (Ehlert et al. 2021).

## Diskussion und Ausblick

Einleitend haben wir die Heterogenität der Zielgruppe der Geringqualifizierten dargestellt und darauf aufbauend eine umfassendere Definition des Begriffs Geringqualifiziert entwickelt, wobei Geringqualifizierung immer als Arbeitsmarktstatus gesehen und in Bezug zu diesem definiert werden muss. Geringqualifizierte, die im Fokus dieses Beitrages stehen, beteiligen sich im Vergleich zu anderen Qualifikationsgruppen seltener an Weiterbildung. Ihre Weiterbildungsbeteiligung ist in den letzten Jahrzehnten zwar stärker als die anderer Qualifikationsgruppen angestiegen, liegt jedoch noch immer hinter jeder anderen Gruppe. Gründe für den Anstieg sind zum einen gestiegene Arbeitsanforderungen und zum anderen Veränderungen des Arbeitsumfelds, bzw. die Einführung neuer Technologien in den Arbeitsprozess. Besonders interessant ist hierbei, dass sich die Weiterbildungsbeteiligung Geringqualifizierter auch nach Kontrolle dieser Faktoren noch von der anderer Qualifikationsgruppen unterscheidet, während sich die Weiterbildungsbeteiligung zwischen anderen Qualifikationsgruppen nach Kontrolle der Faktoren nicht mehr unterscheidet.

Das Ziel des Reviews bestand darin, den empirischen Stand der Weiterbildungsbeteiligung Geringqualifizierter im digitalen Zeitalter und während der Pandemie systematisch zu sichten, um für die Weiterbildungsforschung offene Fragen zu identifizieren. Die recherchierten Artikel des Beitrags weisen tendenziell Beschreibungswissen zum Ist-Zustand auf und liefern wenig Erklärungs- sowie Verbesserungswissen. Außerdem wurden Forschungslücken auf der Ebene des Individuums (kulturelle Faktoren, Familie und Referenzgruppe) und auf der Ebene der Organisation (programmbezogene Merkmale, alternative Programme und Wettbewerb) deutlich. Zukünftige Weiterbildungsforschung sollte sich dementsprechend verstärkt mit Erklärungsmechanismen bezüglich der Weiterbildungsbeteiligung Geringqualifizierter in der Digitalisierung befassen und die Forschung zu programmbezogenen Merkmalen vertiefen. So kann es gelingen, die Zielgruppe der Geringqualifizierten fokussierter mittels Weiterbildungen anzusprechen.

In der betrieblichen Weiterbildung, als größtes Weiterbildungssegment Deutschlands, werden Geringqualifizierte auf zwei Arten sozial selektiert. Erstens bilden Betriebe sozial selektiv weiter, Weiterbildungsangebote richten sich dabei vermehrt an Höherqualifizierte, Führungskräfte und die Stammbelegschaft, dementsprechend erhalten Geringqualifizierte weniger Weiterbildungsangebote (Eichhorst und Schroeder 2019; Matthes und Weber 2019). Zweitens wird ein großer Teil der Geringqualifizierten aufgrund ihrer Erwerbslosigkeit vom größten Weiterbildungssegment Deutschlands sowie von zahlreichen staatlichen Weiterbildungsförderungen (Bildungsgutschein, WeGebAU, QCG und Arbeit-von-morgen-Gesetz) ausgeschlossen. Die vorliegende Arbeit zeigt, dass staatliche Weiterbildungsförderung, wie Bildungsgutscheine, QCG, etc., förderlich für die Weiterbildungsbeteiligung der Gruppe der erwerbstätigen Geringqualifizierten ist (Abb. 2). Insgesamt wurde die Weiterbildungsförderung Schritt für Schritt durch neue Gesetze für weitere Gruppen geöffnet, sodass Geringqualifizierte nicht mehr die Hauptzielgruppe bilden. Dadurch veränderte sich die zielgerichtete Weiterbildungsförderung zu einer Förderung mit der Gießkanne. Wodurch eher Gruppen, die das Lernen gewohnt sind und Weiterbildung positiv gegenüberstehen, partizipieren. Zudem fokussieren die Programme und Gesetze hauptsächlich finanzielle Hürden der Weiterbildungsbeteiligung, dabei weisen Geringqualifizierte auch andere Hürden auf, die fokussiert werden müssten, um deren Weiterbildungsbeteiligung zu steigern. In diesem Kontext benötigt es zielgerichtete Förderprogramme sowie eine gut ausgebaute Beratungsstruktur.

Neben den strukturellen, gesellschaftlichen sowie (förder‑)politischen Faktoren, die die Weiterbildungsbeteiligung Geringqualifizierter beeinflussen, zeigen sich auch auf der Ebene des Individuums und der Organisation Einflussfaktoren. So wirken sich sozioökonomische (Beschäftigungsumfang, berufliche Stellung) und demographische Faktoren (Geschlecht, Alter, Migrationshintergrund) Geringqualifizierter auf die Weiterbildungsbeteiligung aus. Des Weiteren haben eine geringe (Grund‑)Bildung sowie Motivation einen negativen Einfluss. Im Umkehrschluss bildet mangelnde Grundbildung eine Hürde der Weiterbildungsbeteiligung Geringqualifizierter (ebd.; Matthes und Weber 2019). Der Bedarf an Grundbildung wurde auch von staatlicher Seite erkannt, so wurde 2016 die Nationale Dekade für Alphabetisierung und Grundbildung (AlphaDekade) ins Leben gerufen (BMBF 2022). Im Rahmen dessen soll das Grundbildungsniveau der Erwachsenenbevölkerung erhöht werden (ebd.). Auch Unternehmen bieten vermehrt Weiterbildungen zu Grundbildung an, jedoch sind diese meist tätigkeitsorientiert und dienen der Bewältigung des Arbeitsprozesses (Schöpper-Grabe und Vahlhaus 2019). In diesem Kontext wird jedoch auch eine verstärkte Förderung der allgemeinen Grundbildung in Unternehmen benötigt, um zum einen eine Hürde der Weiterbildungsbeteiligung Geringqualifizierter zu adressieren und zum anderen eine Grundlage zur Weiterqualifikation zu bilden und damit zur Beschäftigungssicherheit beizutragen.

Werden die organisationalen Faktoren der Weiterbildungsteilnahme betrachtet, zeigt sich auch hier, dass Geringqualifizierte systematisch ausgeschlossen werden und Betriebe sozial selektiv weiterbilden. Dies ist ein Indiz dafür, dass Geringqualifizierte nicht im Fokus von betrieblichen Weiterbildungen stehen und auch von Seiten der Arbeitgeber wenig Weiterbildungsangebote erhalten. Dies könnte zum Teil auf das Überangebot an geringqualifizierten Arbeitskräften auf dem Arbeitsmarkt zurückzuführen sein, da dadurch auf Seiten der Unternehmen kaum Anreize bestehen, geringqualifizierte Beschäftigte im Gegensatz zu höherqualifizierten Beschäftigten oder der Stammbelegschaft weiterzubilden. Zudem könnte sich die Ansprache der Zielgruppe für Betriebe aufgrund verschiedener Weiterbildungshemmnisse als komplizierter erweisen als bei anderen Qualifikationsgruppen. Positiv wirken hingegen Tarifverträge, ein hoher Organisationsgrad, eine mitarbeiterorientierte Personalpolitik sowie ein hoher Formalisierungsgrad. Auf der Ebene der Bildungseinrichtung wirken sich eine gute bestehende Infrastruktur sowie technische Ausstattung positiv auf die Weiterbildungsbeteiligung aus. Aus der Literaturrecherche geht hervor, dass bislang wenig Evidenz zur Weiterbildungsbeteiligung von Geringqualifizierten in der Digitalisierung auf der Ebene der Organisationen existieren. So lassen sich in den gesichteten Artikeln zwar einige Merkmale der Bildungseinrichtungen finden, allerdings konnten keine programmbezogenen Merkmale festgestellt werden. Insbesondere für die Zielgruppe der Geringqualifizierten können programmbezogene Merkmale relevante Hinweise auf die (Nicht‑)Teilnahme an Weiterbildung liefern, weshalb die Forschung in diesem Bereich vertieft werden sollte.

Die Veränderung des Arbeitsmarktes und folglich die steigenden Arbeits- sowie Kompetenzanforderungen führen zu einem immer größer werdenden Weiterbildungsbedarf von Geringqualifizierten, um diese trotz sich ändernder Bedingungen weiter beschäftigen sowie weiterbilden zu können (Reutter 2017). Steigende Lernbedarfe zeigen sich beispielsweise hinsichtlich Grundbildung, Flexibilität, Kooperations- sowie Kommunikationsfähigkeiten. Zudem werden Medienkompetenzen, also der richtige Umgang mit digitalen Medien, von größerer Bedeutung für die Arbeitsbewältigung (Schöpper-Grabe und Vahlhaus 2019; Seyda 2019b). Trotz voranschreitender Digitalisierung der betrieblichen Weiterbildungslehre schulen Unternehmen Geringqualifizierte im Vergleich zur Gesamtheit der Belegschaft seltener mittels digitaler Medien (Schöpper-Grabe und Vahlhaus 2019). Der Einsatz digitaler Medien könnte sich jedoch aufgrund der zeitlichen und räumlichen Flexibilität positiv auf die Weiterbildungsbeteiligung Geringqualifizierter auswirken, da sich diese besser an individuelle Bedarfe und Lebenslagen anpassen lassen als Präsenzveranstaltungen. Im Feld der Weiterbildungsforschung könnte zukünftig stärker der Frage nachgegangen werden, wie Geringqualifizierte am besten mittels digitaler Medien geschult werden können, um diese Gruppe nicht von Teilen der digitalisierten Weiterbildung auszuschließen. Studien empfehlen einen Blended-Learning-Ansatz mit Lernprozessbegleitern zur Unterstützung und Begleitung des Lernprozesses Geringqualifizierter (ebd.; Seyda 2019b). Auch die Schaffung lernförderlicher Arbeitsplätze könnte sich für die Zielgruppe und deren Weiterbildungsbeteiligung als förderlich erweisen, da lernförderliche Arbeitsplätze höhere oder sich ändernde Arbeitsanforderungen an die Beschäftigten stellen, wodurch die Gelegenheit geboten wird, Neues auszuprobieren und dadurch zu lernen (Seyda 2019b). Zudem haben Beschäftigte die Möglichkeit, gelerntes Wissen anzuwenden, wodurch ein Weiterbildungshemmnis Geringqualifizierter, nämlich der subjektiv empfundene fehlende Bedarf an Weiterbildung, adressiert wird, da durch neue Arbeitsabläufe Lernbedarfe entstehen und gelerntes Wissen während der Arbeit angewendet werden kann (ebd.). Dementsprechend bieten lernförderliche Arbeitsplätze die Gelegenheit neue Kompetenzen zu erwerben sowie den subjektiven Nutzen von Weiterbildung zu erhöhen.

Die COVID-19-Pandemie verschärfte die Ungleichheit der Weiterbildungsbeteiligung zwischen Gering- und Hochqualifizierten auf der Ebene des Individuums sowie auf der Ebene der Weiterbildungseinrichtungen. So wiesen Weiterbildungsanbieter, die sich eher an Höherqualifizierte richten ((Fach‑)Hochschulen, Akademien), einen höheren Realisierungsgrad von Veranstaltungen sowie eine bessere technische Ausstattung auf (Christ et al. 2021). Auch nach dem ersten Lockdown zeigten sich weiterhin Unterschiede zwischen verschiedenen Typen von Weiterbildungsanbietern, somit setzte sich die Ungleichheit zwischen den Qualifikationsgruppen fort (ebd.). Bei der Betrachtung der Nutzung digitaler Weiterbildungsangebote zu beruflichen Zwecken wurde ebenfalls eine Ungleichheit zwischen Gering- und Hochqualifizierten deutlich. Diese ist auf Veränderungen der Arbeitsbedingungen (Homeoffice, Arbeitszeitveränderung, Systemrelevanz) im Zuge der COVID-19-Pandemie zurückzuführen (Ehlert et al. 2021). Um diese Ungleichheit nicht weiter zu verschärfen, sollten auf Seiten der Weiterbildungsanbieter die technischen Mittel angeglichen werden. Schließlich kann nicht ausgeschlossen werden, dass Weiterbildung aus subjektiver Sicht nicht als nützlich oder erforderlich wahrgenommen werden kann, denn Weiterbildung muss nicht per se die Antwort auf ein Problem sein (Holzer 2014). Außerdem sollte nicht ungeachtet bleiben, dass Geringqualifizierte sich „an von aussen gesetzte Anforderungen“ anpassen, um systemkonform zu agieren (Zeuner 2022, S. 18).

Es gibt einige Einschränkungen, die in künftigen Forschungsarbeiten berücksichtigt werden sollten. Zum einen wurden nur die beiden größten deutschen Datenbanken – FIS und Pollux – durchsucht und die Stichwortliste war trotz einer großen Variation an Suchbegriffen möglicherweise nicht vollständig. Zwar wurden 28 Artikel anhand der Suchbegriffe gescreent, dennoch ist es denkbar, dass einige relevante Artikel ausgeschlossen wurden. Eine Recherchestrategie mit einer anderen Fokussierung könnte – muss aber nicht – weitere Befunde liefern. Generell wird durch die Studie deutlich, dass Geringqualifizierte auf verschiedene Weise von Weiterbildung ausgeschlossen werden und dass Weiterbildung auf mehreren Ebenen sozial selektiv ist (Eichhorst und Schroeder 2019; Seyda 2019b). Entgegen den Erwartungen werden viele Tätigkeiten auf dem Helferniveau nicht automatisiert und es werden trotz der Digitalisierung auch weiterhin Tätigkeiten auf dem Helferniveau entstehen (Matthes und Weber 2019). Trotzdem resultieren aus hinzukommenden Technologien und veränderten Arbeitsprozessen neue Weiterbildungsbedarfe für die Zielgruppe, um deren Beschäftigungsfähigkeit zu erhalten.

Aus diesen Gründen halten wir es für notwendig, die Weiterbildungsforschung zu Geringqualifizierten im digitalen Zeitalter zu erweitern, um den sich ändernden Arbeitsbedingungen gerecht zu werden, dem Fachkräftemangel entgegenzuwirken sowie zum Erhalt der Beschäftigungsfähigkeit Geringqualifizierter beizutragen. Angesichts der sich ständig verändernden digitalen Arbeitswelt wird der Frage der Programmplanung für Geringqualifizierte relativ wenig Aufmerksamkeit geschenkt. Diese Frage sollte nicht nur konzeptionell beantwortet, sondern auch empirisch begleitet werden. Die Ergebnisse des Beitrages zeigen, dass insbesondere auf der Organisationsebene noch wenig empirisches Forschungswissen vorhanden ist. Möglich wäre es, Kontextbedingungen von Weiterbildungsorganisationen, Programmplanung, Programmentwicklung und/oder Angebote für Geringqualifizierte im digitalen Zeitalter zu eruieren.

## Footnotes

1 Die umfassende Darstellung der bestehenden Definitionen, der Beziehung zu anderen Begriffen sowie der kritischen Betrachtung des Konzepts „Geringqualifizierte“ findet sich in Lacher et al. (2022).

2 vgl. hierzu https://www.arbeitsagentur.de/arbeitslosengeld-2/ausbildung-weiterbildung-umschulung

## References

[CR1] Autorengruppe Bildungsberichterstattung (2020). Bildung in Deutschland 2020. Ein indikatorengestützer Bericht mit einer Analyse zu Bildung in einer digitalisierten Welt.

[CR2] Bertelsmann Stiftung (2018). Deutscher Weiterbildungsatlas. Teilnahme und Angebot in Kreisen und kreisfreien Städten.

[CR3] Bilger F, Behringer F, Kuper H, Schrader J (2017). Weiterbildungsverhalten in Deutschland 2016. Ergebnisse des Adult Education Survey (AES).

[CR7] BMAS – Bundesministerium für Arbeit und Soziales, & BMBF – Bundesministerium für Bildung und Forschung (2019). Nationale Weiterbildungsstrategie. https://www.bmbf.de/bmbf/shareddocs/downloads/files/nws_strategiepapier_barrierefrei_de.pdf?__blob=publicationFile&v=1. Zugegriffen: 27. Jan. 2022. Studie, die für die Systematisierung der Einflussfaktoren gesichtet wurde [2].

[CR4] BMBF – Bundesministerium für Bildung und Forschung (2021). Weiterbildungsverhalten in Deutschland 2020. Ergebnisse des Adult Education Survey – AES-Trendbericht.

[CR5] BMBF – Bundesministerium für Bildung und Forschung (2022). Monitoring-Bericht. (Zwischen‑)Ergebnisse der vom BMBF im Rahmen der AlphaDekade geförderten Projekte für das Jahr 2020. https://www.alphadekade.de/alphadekade/shareddocs/downloads/files/monitoringbericht-projektergebnisse_2020.pdf.pdf. Zugegriffen: 31. Jan. 2022.

[CR8] Boeren E, Nicaise I, Baert H (2010). Theoretical models of participation in adult education: The need for an integrated model. International Journal of Lifelong Education.

[CR9] Buddeberg K, Stammer C, Dörner O, Iller C, Schüßler I, von Felden H, Lerch S (2020). Schließt der digitale Wandel ältere und gering literalisierte Erwachsene aus?. Erwachsenenbildung und Lernen in Zeiten von Globalisierung, Transformation und Entgrenzung.

[CR10] Bundesagentur für Arbeit (2021). Statistik der Bundesagentur für Arbeit. Grundlagen: Definitionen – Glossar der Statistik der BA.

[CR11] Bundesinstitut für Berufsbildung (2020). *Datenreport zum Berufsbildungsbericht. 12. Datenreport zum Berufsbildungsbericht 2020. Informationen und Analysen zur Entwicklung der beruflichen Bildung* (1. Aufl.). Bonn: Bundesinstitut für Berufsbildung. https://www.bibb.de/dokumente/pdf/bibb_datenreport_2020.pdf. Zugegriffen: 27. Jan. 2022. Studie, die für die Systematisierung der Einflussfaktoren gesichtet wurde [3].

[CR12] Bundesregierung (2021). *Inanspruchnahme der geförderten Weiterbildung nach dem Qualifizierungschancengesetz. Gesetzentwurf der Bundesregierung.* Berlin: Bundestag. http://dipbt.bundestag.de/dip21/btd/19/266/1926648.pdf. Zugegriffen: 27. Jan. 2022.

[CR13] Bußmann S, Seyda S (2016). Fachkräfteengpässe in Unternehmen. Berufe mit Aufstiegsfortbildung: Zwischen Fachkräfteengpässen und Digitalisierung.

[CR14] CEDEFOP – Europäisches Zentrum für die Förderung der Berufsbildung (2018). Zusammenfassung. Investitionen in Kompetenzen zahlen sich aus. https://www.cedefop.europa.eu/files/5560_de_zusammenfassung_0.pdf. Zugegriffen: 21. Okt. 2021.

[CR15] Christ J, Koscheck S, Martin A, Ohly H, Widany S (2021). Auswirkungen der Coronapandemie auf Weiterbildungsanbieter. Ergebnisse der wbmonitor Umfrage 2020.

[CR16] Cross PK (1992). Adults as learners—Increasing participation and facilitating learning.

[CR17] Dengler K, Matthes B, Möller J, Walwei U (2017). Folgen der Digitalisierung für die Arbeitswelt: Welche Berufe sich potenziell durch Computer ersetzen lassen. Arbeitsmarkt kompakt: Analysen, Daten, Fakten.

[CR57] van Dijk J, Bus J, Crompton M, Hildebrandt M, Metakides G (2012). Evolution of the digital divide: the digital divide turns to inequality of skills and usage. Digital enlightenment yearbook.

[CR58] van Dijk J (2020). The digital divide.

[CR18] Ehlert M, Kleinert C, Vicari B, Zoch G (2021). Digitales selbstgesteuertes Lernen Erwerbstätiger in der Corona-Krise. Analysen auf Basis der NEPS-Startkohorte 6.

[CR19] Eichhorst W, Schroeder W, Freise M, Zimmer A (2019). Soziale Innovationen in der Arbeitsmarktpolitik. Zivilgesellschaft und Wohlfahrtsstaat im Wandel.Akteure, Strategien und Politikfelder.

[CR20] Faulstich P (1981). Arbeitsorientierte Erwachsenenbildung.

[CR21] Funken C, Schulz-Schaeffer I (2008). Digitalisierung der Arbeitswelt: zur Neuordnung formaler und informeller Prozesse in Unternehmen.

[CR24] Gollob S, Fleischli M, Sgier I (2021). Auswirkungen der Corona-Pandemie auf die Weiterbildung. Ergebnisse der jährlichen Umfrage bei Weiterbildungsanbietern (Weiterbildungsstudie 2020/2021).

[CR22] Grant MJ, Booth A (2009). A typology of reviews: an analysis of 14 review types and associated methodologies. Health Information & Libraries Journal.

[CR23] Grebe, T., Schüren, V., & Ekert, S. (2017). Evaluation der IHK-Pilotinitiative Zertifizierung von Teilqualifikationen. Abschlussbericht. https://docplayer.org/63646168-Evaluation-der-ihk-pilotinitiative-zertifizierung-von-teilqualifikationen.html. Zugegriffen: 28. Jan. 2022. Studie, die für die Systematisierung der Einflussfaktoren gesichtet wurde [8].

[CR25] Hermeling S, Bolder A, Bremer H, Epping R (2017). „Dann bist du wieder ein Jahr älter und hast immer noch nichts erreicht.“ Die Förderung beruflicher Weiterbildung im Hartz-IV-System. Bildung und Arbeit. Bildung für Arbeit unter neuer Steuerung.

[CR26] Hillmert S, Becker R (2017). Bildung und Lebensverlauf – Bildung im Lebensverlauf. Lehrbuch der Bildungssoziologie.

[CR27] Hirsch-Kreinsen H, Ittermann P, Spöttl G, Windelband L (2017). Drei Thesen zu Arbeit und Qualifikation in Industrie 4.0. Industrie 4.0. Chancen und Risiken für die Berufsbildung.

[CR28] Hofmann, T., Schabram, G., & Rock, J. (2018). Kurzexpertise Nr. 2/2018. Kaum Bildungsaufstieg aus Arbeitslosigkeit. Zur Fort- und Weiterbildung in der Arbeitsförderung. Berlin, *Deutscher Paritätischer Wohlfahrtsverband*. http://infothek.paritaet.org/pid/fachinfos.nsf/0/adeccd23e380132fc12582d600326c9a/$FILE/PaFo-2018-2-FbW.pdf. Zugegriffen: 16. Mai 2022. Studie, die für die Systematisierung der Einflussfaktoren gesichtet wurde [10].

[CR29] Holzer D, Erler I, Holzer D, Kloyber C, Schuster W, Vater S (2014). Weiterbildung ist die falsche Antwort auf falsche Fragen. Eine angedeutete Streitschrift. Wenn Weiterbildung die Antwort ist, was war die Frage?.

[CR30] Hornberg, C., Heisig, J. P., & Solga, H. (2021). Fit für die digitale Arbeitswelt. Weiterbildung gering Qualifizierter scheitert an Strukturen am Arbeitsplatz. *WZB-Mitteilungen* (171), 44–47. https://bibliothek.wzb.eu/artikel/2021/f-23710.pdf. Zugegriffen: 28. Jan. 2022. Studie, die für die Systematisierung der Einflussfaktoren gesichtet wurde [11].

[CR31] IAB Forum – Institut für Arbeitsmarkt- und Berufsforschung der Bundesagentur für Arbeit (2017). Erwerbsfähiges Alter. https://www.iab-forum.de/glossar/erwerbsfaehiges-alter/?pdf=1046. Zugegriffen: 28. Jan. 2022.

[CR32] Käpplinger B, Lichte N (2020). “The lockdown of physical co-operation touches the heart of adult education”: A Delphi study on immediate and expected effects of COVID-19. International Review of Education.

[CR33] Klaus, A., Kruppe, T., Lang, J., & Roesler, K. (2020). Geförderte Weiterbildung Beschäftigter: Trotz erweiterter Möglichkeiten noch ausbaufähig. Paralleltitel: Subsidized training for employed workers: extended possibilities but still room for improvement. Nürnberg*, IAB-Kurzbericht* 24/2020. http://doku.iab.de/kurzber/2020/kb2420.pdf. Zugegriffen: 22. März 2022. Studie, die für die Systematisierung der Einflussfaktoren gesichtet wurde [12].

[CR34] Kleinert, C., Bächmann, A., & Zoch, G. (2020). Erwerbsleben in der Corona-Krise: Welche Rolle spielen Bildungsunterschiede? Analysen auf Basis der NEPS-Startkohorten 2, 4, 5 und 6. Bamberg: LifBi (NEPS Corona und Bildung. 2). https://www.lifbi.de/Portals/13/Corona/NEPS_Corona-und-Bildung_Bericht_2-Erwerbsleben.pdf. Zugegriffen: 22. März 2022. Studie, die für die Systematisierung der Einflussfaktoren gesichtet wurde [13].

[CR35] Kleinert, C., Vicari, B., Zoch, G., & Ehlert, M. (2021). *Wer bildet sich in Pandemiezeiten beruflich weiter? Veränderungen in der Nutzung digitaler Lernangebote während der Corona-Krise.* Bamberg: LifBi (NEPS Corona und Bildung. 7). https://www.lifbi.de/Portals/13/Corona/NEPS_Corona-und-Bildung_Bericht_7-Weiterbildung.pdf. Zugegriffen: 22. März 2022. Studie, die für die Systematisierung der Einflussfaktoren gesichtet wurde [14].

[CR36] Klös H-P (2021). Berufliche Weiterbildung in Deutschland: Status Quo und Weiterentwicklung.

[CR37] Lacher, S., Fliegener, L., & Rohs, M. (2022). Geringqualifizierte. Eine relative Perspektive. *Beiträge zur Erwachsenenbildung, 11*. Technische Universität Kaiserslautern. https://nbn-resolving.org/urn:nbn:de:hbz:386-kluedo-67802. Zugegriffen: 22. März 2022.

[CR38] Lutz C (2019). Digital inequalities in the age of artificial intelligence and big data. Human Behavior and Emerging Technologies.

[CR39] Martin A, Rüber IE (2016). Die Weiterbildungsbeteiligung von Geringqualifizierten im internationalen Vergleich – Eine Mehrebenenanalyse. Zeitschrift für Weiterbildungsforschung.

[CR41] Matthes B, Severing E, Matthes B, Severing E (2017). Berufliche Kompetenzen von Geringqualifizierten erkennen und fördern. Berufsbildung für Geringqualifizierte. Barrieren und Erträge.

[CR40] Matthes B, Weber E, Goth GG, Kretschmer S, Pfeiffer I (2019). Auswirkungen von Digitalisierung und demografischem Wandel für Geringqualifizierte. Bildungsinnovationen für nicht formal Qualifizierte. Zielgruppengerechte Weiterbildungssettings in der Bildungspraxis.

[CR42] Munn Z, Peters MDJ, Stern C, Tufanaru C, McArthur A, Aromataris E (2018). Systematic review or scoping review? Guidance for authors when choosing between a systematic or scoping review approach. BMC Medical Research Methodology.

[CR43] Osiander C, Goth G, Kretschmer S, Pfeiffer I (2019). Zugang zu beruflicher Weiterbildung – Rechtlicher Rahmen, Teilnahme an und Wirkung von beruflicher Weiterbildung, Weiterbildungshemmnisse. Bildungsinnovationen für nicht formal Qualifizierte. Zielgruppengerechte Weiterbildungssettings in der Bildungspraxis.

[CR44] Pfeiffer, I., Dauser, D., Gagern, S., Hauenstein, T., Kreider, I., & Wolf, M. (2019). Weiterbildungsförderung in Deutschland. Bestandsaufnahme und Analyse aktuell genutzter Instrumente. Nürnberg (f-bb-Dossier. 2019,01). https://www.f-bb.de/fileadmin/PDFs-Publikationen/190805_f-bb-Dossier_WB.pdf. Zugegriffen: 18. Dez. 2021. Studie, die für die Systematisierung der Einflussfaktoren gesichtet wurde [17].

[CR45] Ramos CR, Harris R (2012). Training and its benefits for individuals: what form, what for and for whom?. International Journal of Learning.

[CR46] Reutter G (2017). Armut und Erwachsenenbildung. Forum Erwachsenenbildung.

[CR47] Rüber, I. E., & Widany, S. (2021). Gleichstellung durch Weiterbildung in einer digitalisierten Gesellschaft. Stand: Juli 2020. Berlin: Geschäftsstelle Dritter Gleichstellungsbericht der Bundesregierung (Dritter Gleichstellungsbericht der Bundesregierung). https://www.dritter-gleichstellungsbericht.de/kontext/controllers/document.php/126.e/b/759b3b.pdf. Zugegriffen: 18. Dez. 2021. Studie, die für die Systematisierung der Einflussfaktoren gesichtet wurde [18].

[CR48] Schmidt-Hertha B (2021). Die Pandemie als Digitalisierungsschub?. Hessische Blätter für Volksbildung.

[CR49] Schöpper-Grabe S, Vahlhaus I (2019). IW-Trends 1/2019. Grundbildung und Weiterbildung für Geringqualifizierte. Ergebnisse einer IW-Unternehmensbefragung.

[CR67] Schulenberg, W., Loeber, H., Loeber-Pautsch, U., Pühler, S., Driesen, H. & Scharf, W. (1978). *Soziale Faktoren der Bildungsbereitschaft Erwachsener*. Stuttgart: Klett.

[CR50] Seibert H, Wiethölter D, Schwengler B (2021). Beschäftigungsentwicklung von Helfertätigkeiten: Starker Einbruch in der Corona-Krise.

[CR52] Seyda, S. (2019a). *Öffentliche Weiterbildungsförderung stark gestiegen.* Köln: Institut der deutschen Wirtschaft. http://doku.iab.de/externe/2019/k190807v13.pdf. Zugegriffen: 18. Dez. 2021. Studie, die für die Systematisierung der Einflussfaktoren gesichtet wurde [21].

[CR53] Seyda S (2019). Wie die Digitalisierung genutzt werden kann, um Geringqualifizierte weiterzubilden: Handlungsempfehlung an Individuen, Unternehmen und Bildungsanbieter sowie die Bundesagentur für Arbeit.

[CR51] Seyda S, Wallossek L, Zibrowius M (2018). Keine Ausbildung – keine Weiterbildung? Einflussfaktoren auf die Weiterbildungsbeteiligung von An- und Ungelernten.

[CR54] Sperber, C., & Walwei, U. (2017). Entwicklung und Struktur der Beschäftigungsverhältnisse. In: J. Möller, U. Walwei (Hrsg.): *Arbeitsmarkt kompakt: Analysen, Daten, Fakten*. (S. 38–40). Bielefeld: Wbv. https://www.ssoar.info/ssoar/handle/document/64169. Zugegriffen: 18. Dez. 2021.

[CR55] Statistisches Bundesamt (Destatis), Wissenschaftenzentrum Berlin für Sozialforschung (WZB), Bundesinstitut für Bevölkerungsforschung (BiB) (2021). Datenreport 2021. Ein Sozialbericht für die Bundesrepublik Deutschland.

[CR56] Umsetzungsbericht der NWS in: BMAS – Bundesministerium für Arbeit und Soziales; BMBF – Bundesministerium für Bildung und Forschung (2021). Umsetzungsbericht Nationale Weiterbildungsstrategie. https://www.bmbf.de/bmbf/shareddocs/downloads/files/nws_strategiepapier_barrierefrei_de.pdf?__blob=publicationFile&v=1. Zugegriffen: 18. Dez. 2021. Studie, die für die Systematisierung der Einflussfaktoren gesichtet wurde [24].

[CR59] Van Nieuwenhove L, De Wever B (2021). Why are low-educated adults underrepresented in adult education? Studying the role of educational background in expressing learning needs and barriers. Studies in Continuing Education.

[CR60] Wiß T (2017). Employee representatives’ influence on continuing vocational training. The impact of institutional context [Der Einfluss von Arbeitnehmervertretern auf die Weiterbildung. Die Auswirkung institutioneller Faktoren]. European Journal of Industrial Relations.

[CR61] Wolf, M., Hecker, K., Kohl, M., & Pfeiffer, I. (2018). Konzepte modularer Nachqualifizierung: Hintergrund, aktuelle Entwicklungen und praktische Anwendung. https://www.pedocs.de/volltexte/2020/18536/. Zugegriffen: 18. Dez. 2021. Arbeitspapier im Rahmen der Workshops „Schritt für Schritt zum Berufsabschluss“. Rahmenbedingungen und Umsetzung von Teilqualifizierungen in der beruflichen Bildung. Nürnberg: Forschungsinstitut Betriebliche Bildung (f-bb). Studie, die für die Systematisierung der Einflussfaktoren gesichtet wurde [26].

[CR63] Wotschack P (2017). Unter welchen Bedingungen bilden Betriebe an- und ungelernte Beschäftigte weiter? Eine institutionentheoretische Untersuchung auf Basis von Daten des IAB-Betriebspanels: Paralleltitel: When do companies train unskilled workers? An institutional study based on data from the IAB establishment survey. Zeitschrift für Soziologie.

[CR64] Wotschack P (2020). When do companies train low’skilled workers? The role of institutional arrangements at the company and sectoral level [Wann erhalten geringqualifizierte Arbeitnehmer eine betriebliche Weiterbildung? Die Rolle institutioneller arrangements auf Ebene des Betriebs und des Sektors]. BJIR: An International Journal of Employment Relations.

[CR62] Wotschack P, Solga H (2014). Betriebliche Weiterbildung für benachteiligte Gruppen. Förderliche Bedingungskonstellationen aus institutionentheoretischer Sicht. Berliner Journal für Soziologie.

[CR65] Zeuner C (2022). Gesellschaftlicher Bildungsbedarf und subjektive Bildungsbedürfnisse: Perspektivverschränkungen. Education Permanente EP.

[CR66] Ziegler P, Akbar S (2021). Gering Qualifizierte als Quelle zur unternehmensinternen Deckung des Fachkräftebedarfs – Good-Practice-Recherche in ausgewählten europäischen Ländern. Projektabschlussbericht des Wiener Instituts für Arbeitsmarkt- und Bildungsforschung (WIAB).

[CR68] Barz, H., & Tippelt, R. (2003). Bildung und soziales Milieu: Determinanten des lebenslangen Lernens in einer Metropole. *Zeitschrift für Pädagogik*, *49*(3), 323-340.

